# Efficacy of a novel antimicrobial hydrogel for eradication of *Staphylococcus epidermidis*, *Staphylococcus aureus* and *Cutibacterium acnes* from preformed biofilm and treatment performance in an *in vivo* MRSA wound model

**DOI:** 10.1093/jacamr/dlab108

**Published:** 2021-07-29

**Authors:** Troels Ronco, Maria F Aragao, Søren Svenningsen, Jørn B Christensen, Anders Permin, Lasse Saaby, Nina Bionda, Ellen E Lantz, Rikke H Olsen

**Affiliations:** 1 Department of Veterinary and Animal Sciences, Faculty of Health and Medical Sciences, University of Copenhagen, Copenhagen, Denmark; 2 Department of Chemistry, Faculty of Science, University of Copenhagen, Copenhagen, Denmark; 3 Unibrains, Virum, Denmark; 4 Bioneer: FARMA, Department of Pharmacy, Copenhagen, Denmark; 5 iFyber, New York, NY, USA

## Abstract

**Background:**

Bacterial biofilm formation is a complicating factor in the antimicrobial treatment of bacterial infections.

**Objectives:**

In this study, we assessed the impact of a novel hydrogel with the active antimicrobial compound JBC 1847 on eradication of preformed biofilms of *Staphylococcus epidermidis*, *Cutibacterium acnes* and MRSA *in vitro*, and evaluated the *in vivo* efficacy of MRSA wound treatment.

**Methods:**

Biofilms were exposed to JBC 1847 for 24 h and subsequently the treatments were neutralized and surviving biofilm-associated bacteria recovered and enumerated. The efficacy of the hydrogel on post-treatment load of MRSA was determined in a murine model of MRSA wound infection, and skin samples of the infected mice were examined histologically to evaluate the degree of healing.

**Results:**

A concentration-dependent eradication of biofilm-embedded bacteria by JBC 1847 was observed for all three pathogens, and the hydrogel caused a greater than four log reduction of cfu in all cases. In the mouse model, treatment with the hydrogel significantly reduced the cfu/mL of MRSA compared with treatment of MRSA-infected wounds with pure hydrogel. Histopathological analysis of the wounds showed that the JBC 1847 treatment group had a lower grade of inflammation, a higher mean score of re-epithelization and higher mean scores of parameters assessing the maturity of the newly formed epidermis, compared with both the fusidic acid 2% and vehicle treatment groups.

**Conclusions:**

The novel hydrogel shows promising results as a candidate for future wound treatment, likely to be highly effective even in the case of biofilm-complicating infected wounds.

## Introduction

Antimicrobial resistance is a considerable threat to the public health worldwide.^1^ Moreover, biofilm formation is a complicating factor that makes it difficult to treat bacterial infections. Biofilms are extracellular polymeric matrices that have been shown to be more resilient to antimicrobial treatment compared with planktonic cells.^2^^,^^3^*Staphylococcus epidermidis*, *Staphylococcus aureus* and *Cutibacterium acnes* are well-known biofilm producers and cause a variety of infections, such as skin, soft tissue and persistent implant-associated infections.^4^^,^^5^ Since the number of novel antimicrobials developed within the last decade has been decreasing remarkably, there is an urgent need for novel clinical treatment solutions.^6^ Therefore, we have evaluated various previously synthesized phenothiazine derivatives that have exhibited efficient antimicrobial activity.^7^^,^^8^ The most promising compound is JBC 1847, a promazine derivative, which has shown strong antimicrobial activity both *in vitro* and *in vivo* against *S. aureus*.^8^ In addition, our previous study also suggested that *S. aureus* do not develop rapid tolerance to JBC 1847 compared with fusidic acid, the latter of which previously has been reported to constitute a considerable clinical issue due to the development of high tolerance in *S. aureus*.^9^^,^^10^

The main aim of this study was to investigate the biofilm eradication efficacy of JBC 1847 hydrogel formulation against preformed *S. epidermidis*, *S. aureus* and *C. acnes* biofilm, as well as examine the extent of inhibition of biofilm formation. Furthermore, we wanted to investigate the efficiency (post-treatment *S. aureus* reduction and wound healing) of the antimicrobial hydrogel in an *S. aureus in vivo* wound model.

## Methods

### Ethics

All animal procedures were carried out at Statens Serum Institut (SSI), Copenhagen, Denmark, and approved under a Danish Animal Experiment Inspectorate. SSI has an Animal Welfare Committee that is equivalent to IACUC (American Association for Laboratory Animal Science) and provides general guidelines for all animal experiments conducted at SSI. Therefore, we declare that all ethical principles and guidelines for experiments on animals were carefully followed.

### Synthesis of JBC 1847 and preparation of JBC 1847-containing hydrogel

Synthesis of JBC 1847 was done at University of Copenhagen, according to Ronco *et al.*^8^ A hydrogel-based formulation of JBC 1847 was prepared with hydroxypropyl methylcellulose (3% w/v, Methocel K200M, Colorcon Ltd, Dartford, Kent, UK) and polyethylene oxide (Polyox WSR 205, Colorcon Ltd) in modified Dulbecco’s PBS (DPBS), without calcium chloride and magnesium chloride (Sigma–Aldrich, D8537, St Louis, MO, USA). Briefly, JBC 1847 (2% w/v) was suspended in ice cold modified DPBS under vigorous stirring. Subsequently, Methocel K200M (3% w/v) and Polyox WSR 205 (3% w/v) were slowly added to the suspension of JBC 1847, while maintaining vigorous stirring. The partially hydrated gel was stored overnight at 4°C to complete the hydration of the gel.

### Determination of MIC and MBC of JBC 1847

The antimicrobial activity of JBC 1847 (in saline solution) was evaluated for the three Gram-positive strains: *S. epidermidis* 35984, *C*. *acnes* 6919 and *S. aureus* USA300. The MIC assay protocol followed standard CLSI guidelines for the broth microdilution assay listed in document ‘Methods for Dilution Antimicrobial Susceptibility Tests for Bacteria that Grow Aerobically: M07-A10’^11^ for *S. epidermidis* and *S. aureus*, and guidelines according to the document ‘Methods for Antimicrobial Susceptibility Testing of Anaerobic Bacteria: M011-A6’^12^ for *C. acnes.*

For the MBC assay, planktonic bacteria were mixed in equal volumes with 2-fold serial dilutions of test compounds in a 96-well challenge plate, with a final volume of 0.2 mL/well. Final concentrations of test compounds ranged from 16–0.125 mg/L in CAMHB with 0.1% DMSO. Dilution of overnight cultures of *S. epidermidis* and *S. aureus* yielded levels of about 2 × 10^5^ cfu/mL in assay wells. The challenge plate also included growth control wells without JBC 1847, as well as sterility control wells without bacteria, which served as a spectrophotometric blank. All samples were evaluated in triplicate. After incubation in a stationary incubator for 24 h at 37°C, wells were evaluated for microbial growth using a spectrophotometric readout at 650 nm. Representative aliquots of all samples were then transferred to a separate 96-well plate containing Dey–Engley (D/E) broth to neutralize the test compound. D/E broth is a general neutralizer for a range of antimicrobial agents and it’s used to ensure that the observed reduction in microbial growth is indeed due to the effects of the treatment during the assay, not due to the effect of leftover treatment during the enumeration procedure (incubation of bacteria on agar overnight).^13^^,^^14^ To perform neutralization and enumeration, 20 μL aliquots of samples from the MIC plate were transferred to a new 96-well plate containing 180 μL of D/E in the first row. For a visual comparison of growth after neutralization, 10 μL from each well was spot plated on a large 1.5% tryptic soy agar (TSA) plate, incubated for 24 h at 37°C, and scanned. Surviving bacteria were also enumerated by spot plating 10-fold serial dilutions of the neutralized treatments (3 × 10 μL for each dilution) on 1.5% TSA. Undiluted neutralized samples were also spread plated (100 μL) on 1.5% TSA as needed. After sufficient growth, plates were counted, and data are reported as log_10_ cfu/mL of surviving bacteria. MBC determination of *C. acnes* was conducted as described above, except a 48 h culture was diluted 1/500 in CAMHB and the challenge plate was incubated anaerobically for 48 h before neutralization and enumeration. The initial level of bacteria in the challenge plate was approx. 1 × 10^6^ cfu/mL

### Eradication of preformed biofilms in vitro

Bacterial biofilms were grown on 96-well plates. Overnight cultures of *S. epidermidis* 35984 and *S. aureus* USA300 or 48 h cultures for *C*. *acnes* 6919 were diluted 1:20 in tryptic soy broth (TSB) and 100 μL was added to interior wells of the plate, while 100 μL of TSB was added to perimeter wells for humidity. In an appropriate atmosphere, plates were incubated at 37°C for 24 h in a stationary incubator. The inocula were about 2 × 10^7^ cfu/mL for *S. epidermidis* and *S. aureus* and 2 × 10^6^ cfu/mL for *C. acnes*.

Next, the biofilm wells were aspirated and briefly rinsed thrice with 200 μL PBS to remove media and planktonic bacteria, then treated with 200 μL JBC 1847 per well. The stock solution of JBC 1847 was diluted to 1 ×, 4 × and 10 × the MIC in CAMHB with 1% DMSO and 200 μL was added to each well. The hydrogel of JBC 1847 was added with a syringe. CAMHB with 1% DMSO was added to the growth control column (served as untreated control) and CAMHB was added to remaining wells. All treatment conditions, as well as controls, were evaluated in six sample replicates and each experiment was performed twice. The treated plate was incubated in an appropriate atmosphere in a stationary incubator at 37°C for an additional 24 h.

After 24 h, the treatments were neutralized and the surviving biofilm-associated bacteria recovered and enumerated. All wells in the plate were aspirated, relevant wells were neutralized with 300 μL broth (D/E) for 10 min, D/E was then aspirated and replaced with fresh 300 μL D/E. The plate was sonicated for 20 min by placing it on a metal lid on top of a sonicator bath. Bacterial survivors were enumerated as described above. After appropriate incubation, colonies were counted, and results given as microbial survival in log_10_ cfu/mL.

### Prevention of MRSA biofilm formation in vitro

In addition to assessing the efficacy of JBC 1847 to eradicate bacteria from preformed biofilms (previously described), the efficacy to prevent MRSA biofilm formation was also evaluated. Prior to initiating the biofilm prevention studies, it was first evaluated whether the neutralization protocol was adequate for JBC 1847. To do this, an overnight culture of MRSA USA300 was diluted 1:100 000 in D/E. This inoculum was then spiked with JBC 1847 in a 1:20 ratio—the final concentration was 8 mg/L. The samples were left to sit at room temperature for 10 min followed by quick vortexing and spread plating of 200 μL of each sample on individual 1.5% TSA plates. The experiment was evaluated in triplicate along with the control, which received only sterile water. Neutralization was deemed successful if the number of cfu for samples with JBC 1847 was 70%–130% of the cfu for the controls. For the biofilm inhibition assay, 96-well polystyrene plates were inoculated with 6 × 10^4^ cfu/mL of MRSA USA300 in CAMHB and challenged with a range of concentrations 0–8 mg/L of JBC 1847, in 2-fold serial dilution fashion. The plates were incubated for 8 h in stationary conditions. Each concentration was evaluated using six sample replicates. Following the incubation time, the spent medium was aspirated, and the wells washed with D/E broth (300 μL/well). The D/E medium was aspirated and a fresh 300 μL added to each well. The plates were then sonicated for 20 min by placing them on a metal lid on top of a sonicator bath. Each sample was then plated for enumeration and the data are reported as log_10_ cfu/mL.

### In vivo treatment of MRSA infected wound

The purpose of this study was to investigate the effect of the JBC 1847 hydrogel against MRSA 43484 (clinical USA300 isolate from a skin infection), in a murine skin infection model,^15^ measured as cfu reduction and post-treatment wound healing. Treatment was performed twice daily for 3 days. Treatment with fusidic acid 2% (Fucidin^®^, Leo Pharma, Ballerup, Denmark) was included as a positive control and treatment with vehicle (hydrogel without JBC 1847) was included as a negative control.

The study was conducted as described by Ronco *et al*.^8^ Briefly, approx. 1 h before inoculation, mice (52 BALB/c female mice, 18–22 g, 8–12 weeks of age, Taconic, Denmark) were treated orally with 45 mL Nurofen (20 mg ibuprofen/mL corresponding to approx. 30 mg/kg) as pain relief. The mice were anaesthetized with 0.15 mL subcutaneously of Zoletil mix. The fur was removed from a 2 × 3 cm area on the back of each mouse using an electric shaver. Next, a razor was used to remove all the hair and thereafter the outer most layer of the skin was scraped off with a dermal curette to obtain a 1 cm^2^ superficial skin lesion. Inoculum (10 μL) containing approx. 1 × 10^7^ cfu of MRSA 43484 was spread on the lesion. After the applied inoculum had dried, the mouse was placed in the cage and kept in a warming cabinet until fully awake.

Before the start of treatment, five mice were sacrificed to determine the average cfu per wound before treatment. Topical treatment was initiated the day after inoculation, Day 1. A total of 16 mice were assigned to each treatment group [JBC 1847 hydrogel (20 mg/g), fusidic acid (20 mg/g) or vehicle group, respectively]. Mice were treated twice daily (9 am and 3 pm) for 3 days. A volume of 50 μL was spread on the inoculated skin area. In each group, half of the mice were sacrificed at Day 4 post-infection (p.i.) to determine cfu/wound/mouse, while the remaining eight mice were sacrificed 7 days p.i. in order to perform histopathological evaluation of wound healing.

The affected skin area was removed by a pair of scissors and tweezers and collected on Day 1 [four mice included in start-of-treatment (SOT) group], Day 4 (8 mice per group) in a tube for a Dispomixer with 1 mL saline or Day 7 (8 mice per group) in 4% buffered formalin. For cfu determination the skin sample was homogenized in a Dispomixer. Each sample was serially diluted in saline/Triton-x and 20 μL spots were applied on MRSA Brilliance agar plates. All agar plates were incubated for 20–48 h at 35°C. The skin samples in formalin were examined histopathologically at Charles River Laboratories (France). The samples were routinely embedded in paraffin and one central section was prepared from each wounded skin site, mounted on a glass slide and stained with haematoxylin and eosin. Histopathological qualitative and semi-quantitative evaluation on local tissue effects (number of inflammatory cells) and healing performance was conducted, based on an adaptation of the ISO 10993 guideline, part 6.

Statistical analysis [analysis of variance (ANOVA)] was performed using GraphPad Prism version 9.0.2 (GraphPad Software, San Diego, CA, USA).

## Results and discussion

### JBC 1847 has bactericidal activity against S. epidermidis, S. aureus and C. acnes in preformed biofilm and highly decreases MRSA biofilm formation

Both MIC and MBC were in the range of 1–4 mg/L for the three species under investigation (Table 1). The similar values of MIC and MBC are indicative of the potent bactericidal activity of JBC 1847. Overall, there is no evidence that bactericidal antimicrobials should be preferred over bacteriostatic antibiotics in topical treatment, e.g. bacteriostatic antibiotics such as clindamycin and erythromycin are frequently used in the clinical treatment of severe acne whereas tetracycline is less frequently applied.^16^ There is, however, accumulating evidence that bactericidal antibiotics reduce the bacterial resistance mechanisms. This is in agreement with previous findings that resistance over time develops 200 times slower in *S. aureus* exposed to JBC 1847 (23 days continuous exposure with daily sub-culturing) than fusidic acid, which is considered to be bacteriostatic against most pathogens.^17^ It should be noted that some topical antimicrobials, such as mupirocin, have a concentration-dependent mode of action, being bacteriostatic at low concentrations but bactericidal in the concentrations obtained at topical treatment.^18^

**Table 1.** MIC and MBC of JBC 1847 for three Gram-positive bacterial species

Species	Strain	MIC (mg/L)	MBC (mg/L)
*S. epidermidis*	35984	2	2
*C. acnes*	6919	1	4
*S. aureus*	USA300	2	4

**Figure 1.**
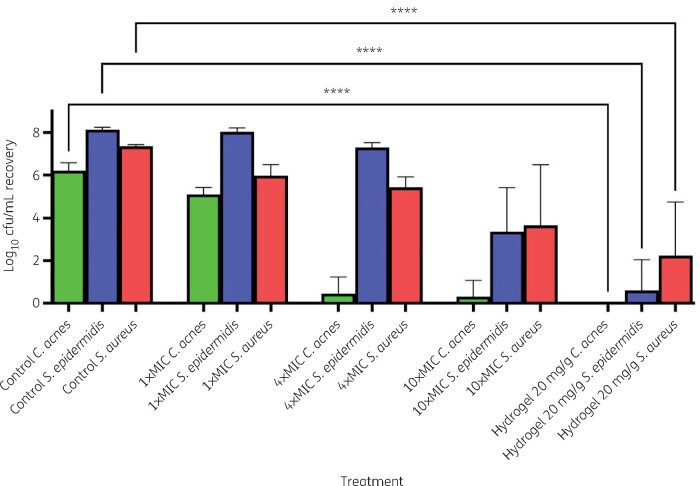
Efficacy of JBC 1847 in eradicating preformed *S. epidermidis* 35984, *S. aureus* USA300 and *C. acnes* 6919 biofilms *in vitro*. The graph shows the total log_10_ cfu of *S. epidermidis*, *S. aureus* and *C. acnes* in recovery solution after 24 h of treatment with JBC 1847 in various concentration (1 ×, 4 × and 10 × MIC) in saline solution or hydrogel solution 20 mg/g. ****P<0.0001.

Concentration-dependent eradications of *S. epidermidis*, *C. acnes* and *S. aureus* by treating preformed *in vitro* biofilms with JBC 1847 were observed (Figure 1). JBC 1847-hydrogel treatment of biofilm-embedded bacteria completely eradicated all *C. acnes*, while less than 10 out of 10^8^ cfu/mL of *S. epidermidis* survived the treatment (no surviving isolates of *S. epidermidis* could be recovered from the biofilm post-treatment). This finding is of particular interest, as biofilm-producing bacteria present unique challenges for effective antimicrobial treatments.^19^ It is worth noting that although the MIC values for *C. acnes* and the two strains of staphylococci differed only by 1-fold (1–2 mg/L, Table 1), *C. acnes* in preformed biofilm could be significantly decreased at only 4 × MIC, whereas both strains of staphylococci were significantly reduced from the preformed biofilm at much higher concentrations (Figure 1). This could be due to differences in the properties of biofilms formed by staphylococci and *C. acnes*. To completely explain the observed difference between the JBC 1847 activity on the two genera when present in preformed biofilms would require more detailed studies, which would be beyond the scope of the present study.

**Figure 2.**
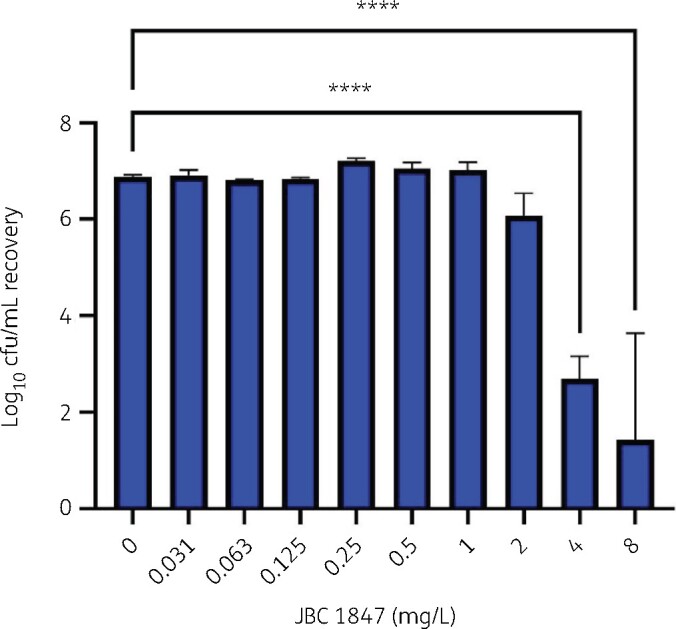
Recovery of MRSA USA300 (log_10_ cfu/mL) in a biofilm inhibition assay. ****P<0.0001.

As shown in Figure 2, JBC 1847 also displays a concentration-dependent inhibition of biofilm formation for MRSA USA300. For JBC 1847, at 4 mg/L there were about 2 log fewer biofilm-associated bacteria compared with untreated controls. An increase of the concentration to 8 mg/L showed an additional increase in efficacy, though not complete inhibition of biofilm formation. While this is an *in vitro* model for biofilms, it is considered a useful tool that provides relevant information with respect to antibiofilm potential of compounds and is routinely used in screening prior to *in vivo* testing.^20^^,^^21^

Although JBC 1847 did not completely inhibit biofilm formation, other studies have showed that subinhibitory concentrations of mupirocin, an antibiotic commonly used in wound therapy, even *promoted* biofilm formation of *S. aureus*, in particular the MRSA USA300 clone.^22^ Similarly, only a limited reduction of biofilm-embedded bacteria (under 25%) has been reported for fusidic acid.^22^ In these aspects, the results obtained for JBC 1847 regarding biofilm inhibition and biofilm-bacterial eradication properties, at least in the evaluated collection of strains, are highly encouraging.

**Figure 3.**
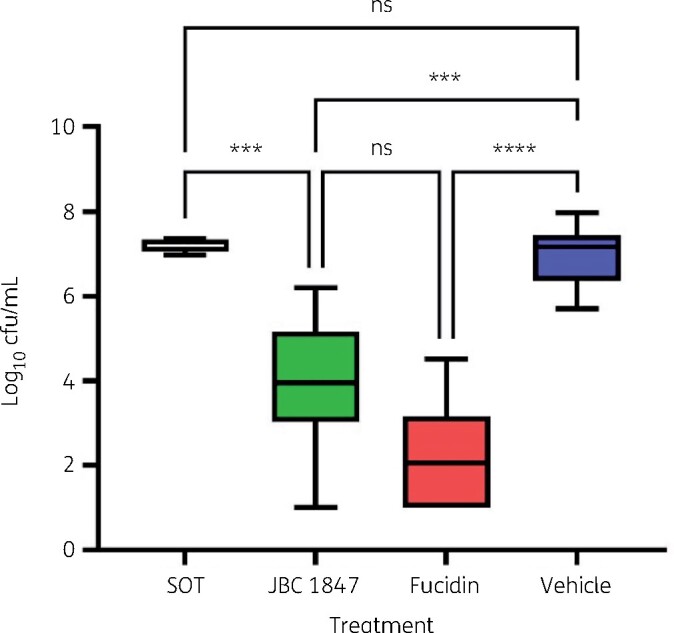
MRSA (cfu/mL) at SOT (Day 1) and after 3 days of topical treatment (twice daily) with JBC 1847 hydrogel, fusidic acid 2% or vehicle (pure hydrogel) in a superficial skin model. ns, not significant; ***P<0.001; ****P<0.0001.

In the *in vivo* experiment the colony count in the inoculum of MRSA 43483 was determined to be 8.88 log_10_ cfu/mL, corresponding to 6.88 log_10_ cfu/mouse (Figure 3). Colony counts in skin lesions were performed at Day 1 (SOT group) and Day 4 post-inoculation. The cfu counts were log_10_ transformed before performing calculations. No mice showed any clinical signs of infection or distress during the study. Treatment with JBC 1847 formulation resulted in a significant (*P *=* *0.0002; ANOVA; multiple comparisons) reduction of the bacterial loads of 3.1 log_10_ cfu compared with vehicle treatment in the skin lesion. Treatment with fusidic acid 2% resulted in a 4.8 log_10_ reduction of the cfu levels compared with the vehicle (hydrogel) control (*P *<* *0.0001) (Figure 3). The reduction of cfu in the fusidic acid 2% treatment was considerably lower than was previously reported using this model,^8^ yet there were no significant differences in mean bacterial load in the JBC 1847 and fusidic acid 2% treatment group. As expected, the hydrogel composition itself did not possess any antimicrobial activity. No mice in either of the three groups showed any clinical signs of systemic infection or distress during the study in either of the treatment groups. Skin samples obtained 4 days after the last treatment day (Day 7 p.i.) were evaluated histologically. Microscopic observations of the wound sites allowed adequate evaluation of the six sites in the JBC 1847 treatment group, and eight sites in each of the vehicle and fusidic acid 2% groups. The wound sites from two animals were excluded from the analysis because they were either incomplete or not in the wound centre in spite of histological recuts. For all remaining sites, tissue margins were present in sufficient amounts to allow evaluation of the entire initial wound. The extent (width and depth) of the wound sites showed good intergroup homogeneity.

**Figure 4.**
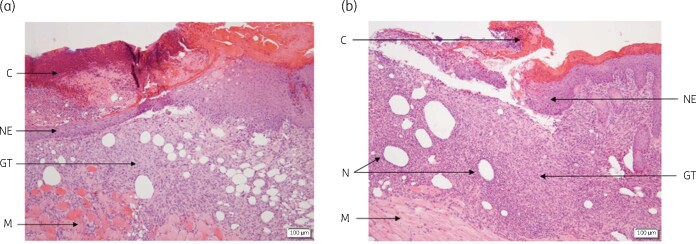
Histopathological section of wounded skin samples from a JBC 1847-treated mouse (a) and a vehicle-treated mouse (b) stained with haematoxylin and eosin. C, crust; GT, granulation tissue; N, aggregates of degenerated neutrophils; M, muscle; NE, newly formed epidermis; SC, subcutis. Notice the larger number of aggregates of degenerated neutrophils and less newly formed epidermis in the slide from vehicle-treated mouse compared with the slide from the JBC 1847-treated mouse.

Overall, the wounds were almost completely to completely epithelialized, with the newly-formed epidermis being thick, not fully differentiated and non-tightly adherent to the dermis (Figure 4). The healing connective tissue corresponded to a mature granulation tissue, characterized by generally abundant fibroblasts, moderate numbers of newly formed vessels (neovascularization) and moderate collagen deposits. The inflammatory cell infiltrate was mainly composed of macrophages and neutrophils (polymorphonuclear cells), as well as fewer lymphocytes and plasma cells. While many polymorphonuclear cells had a normal morphology, some neutrophils appeared degenerated, as characterized by hyper-segmented or fragmented nuclei. They formed multifocal aggregates in the granulation tissue, frequently centred on optically empty large vacuoles consistent with subcutaneous adipocytes. Small round basophilic microorganisms consistent with bacteria were occasionally observed close to degenerate neutrophils. When comparing the three groups in terms of local tissue effects, the JBC 1847 group had lower mean scores of polymorphonuclear cells, macrophages and degeneration (degenerate neutrophils) than the vehicle and fusidic acid 2% treatment groups (Table 2). There were no relevant differences in other parameters. These results indicate a slightly lower grade of inflammation in wound sites of the JBC 1847 group. In terms of performance, the overall wound healing mean score was slightly higher in the test group, with higher mean scores of re-epithelialization (assessing the wound closure), and slightly higher mean scores of parameters assessing the maturity of the newly-formed epidermis (epidermal differentiation and indentation, adherence to the dermis). There were also slightly higher mean scores of parameters assessing the maturity of the healing connective tissue (fibroblasts and collagen deposits), even though there were no relevant differences in the neovascularization mean scores. No material (foreign body) residues were identified. These results indicate a slightly higher healing performance in the JBC 1847 group. Of note, the vehicle (hydrogel) group had a slightly higher grade of inflammation (higher mean scores of polymorphonuclear cells, macrophages and degenerate neutrophils) and lower overall healing mean score when compared with the reference group.

**Table 2.** Semi-quantitative histopathologic analysis

Group	Local tissue effects										Performance								
	polymorphonuclear cells	lymphocytes	plasma cells	macrophages	giant cells	necrosis	haemorrhage	fibrin	degeneration		neovascularization	fibroblasts	collagen deposits	re-epithelialization	epidermal differentiation	adherence to the dermis	epidermal indentation	material residue	overall wound healing
JBC 1847 (*n *=* *6)																			
mean	2.0	1.0	1.0	2.0	0.0	0.0	0.5	0.3	0.5		2.0	3.2	3.0	3.5	2.2	2.7	1.5	0.0	9.7
SD	0.0	0.0	0.0	0.0	0.0	0.0	0.5	0.5	0.5		0.0	0.4	0.0	0.8	0.4	0.9	0.5	0.0	0.7
Vehicle (*n *=* *8)																			
mean	2.9	1.0	1.0	2.9	0.0	0.0	0.3	0.4	1.6		1.9	2.9	2.1	2.8	2.0	2.1	1.0	0.0	7.6
SD	0.3	0.0	0.0	0.3	0.0	0.0	0.4	0.5	0.9		0.3	0.3	0.3	0.8	0.0	1.3	0.0	0.0	1.0
Fusidic acid (*n *=* *8)																			
mean	2.4	1.0	1.0	2.3	0.0	0.0	0.4	0.3	1.1		2.1	3.0	2.6	3.3	1.9	2.6	1.1	0.0	8.8
SD	0.5	0.0	0.0	0.4	0.0	0.0	0.5	0.4	0.8		0.3	0.0	0.5	1.0	0.3	0.5	0.3	0.0	0.8

The table shows semi-quantitative histopathologic analysis of MRSA-infected wound exposed to 3 days of treatment with either JBC 1847, fusidic acid or vehicle. The skin samples were collected 4 days after the last treatment day. *n* indicates the number of analysed sites per group; mean is the mean score calculation.

In conclusion, these initial investigations of a novel JBC 1847-containing hydrogel showed promising results in clearing infected wounds, and the subsequent healing of the wounds was similar to or slightly better than wounds in the fusidic acid 2% treatment group. Of importance, there was an efficient eradication of biofilm-embedded bacteria by the antimicrobial hydrogel, which is encouraging for potential treatment of profound wounds infiltrated with bacterial biofilm. Further evaluations on toxicity, skin permeability and skin-allogenic potential are now required to fully conclude on the clinically application of JBC 1847.
